# Limb salvage for an aorto-bifemoral vascular graft infection using multilayer negative pressure wound therapy: a case report

**DOI:** 10.1097/RC9.0000000000000998

**Published:** 2026-09-14

**Authors:** Giorgio Caddia, Andrea Magi, Salvatora Dettori, Chiara Santoro, Giulio J. Jaber, Corrado Rubino

**Affiliations:** a Plastic and Reconstructive Surgery Unit, Department of Surgery, Azienda Ospedaliero-Universitaria di Sassari, Sassari, Italy; b Vascular and Endovascular Surgery Unit, Department of Cardiovascular Sciences, Azienda Ospedaliero-Universitaria di Sassari, Sassari, Italy; c Plastic and Reconstructive Surgery Unit, Department of Surgery, Azienda Ospedaliero-Universitaria Policlinico “G. Martino”, Messina, Italy; d Department of Plastic and Reconstructive Surgery, IEO – Istituto Europeo di Oncologia, IRCCS, Milan, Italy; e Department of Medicine, Surgery and Pharmacy, University of Sassari, Sassari, Italy

**Keywords:** exposed vascular graft, limb salvage, negative pressure wound therapy, vascular graft infection, wound healing

## Abstract

**Introduction:**

Exposure and infection of aorto-bifemoral grafts are severe complications associated with a high risk of catastrophic hemorrhage and limb loss.

**Case presentation:**

A 71-year-old man presented with rupture of a femoral anastomotic pseudoaneurysm after previous aorto-bifemoral bypass. Following emergency vascular reconstruction, extensive debridement resulted in a large groin defect with exposure of the vascular graft and distal anastomotic sites, complicated by infection and multi-organ failure. In the absence of viable reconstructive options, low-pressure negative-pressure wound therapy (NPWT) was initiated using a customized three-layer protective interface incorporating a silver-impregnated non-adherent dressing placed directly over the graft. Progressive granulation tissue formation allowed staged wound closure and complete healing without flap reconstruction. At follow-up, no clinical evidence of recurrent infection was observed.

**Clinical discussion:**

NPWT over exposed vascular grafts remains controversial because of the risk of catastrophic hemorrhage. In this patient, extensive soft-tissue destruction and unfavorable vascular anatomy created a true “no-option” reconstructive scenario, making low-pressure NPWT followed by staged wound closure the only feasible limb- and graft-preserving strategy. The customized three-layer protective interface was designed to combine local antimicrobial activity with mechanical protection of the exposed graft.

**Conclusion:**

This case suggests that a tailored three-layer NPWT protective interface incorporating a silver-impregnated dressing may represent a useful adjunct to optimize local infection control in exposed vascular grafts. In carefully selected patients without reconstructive options, low-pressure NPWT followed by staged wound closure may represent a valuable limb- and graft-preserving strategy.

## Introduction

Exposure of an aorto-bifemoral graft in the groin represents one of the most severe complications in vascular surgery. It is estimated that approximately 6% of these procedures develop postoperative prosthetic infections^[^1^]^. These events are associated with a high risk of catastrophic hemorrhage from anastomotic disruption and significant morbidity and mortality. Standard management traditionally includes graft removal and soft-tissue coverage using flaps^[^2^]^.

HIGHLIGHTS

- A customized three-layer negative-pressure wound therapy (NPWT) interface incorporating a silver-impregnated dressing was applied directly over an exposed and infected vascular graft.
- Extensive tissue loss and compromised vascular anatomy created a true “no-option” reconstructive scenario.
- Low-pressure NPWT served as a bridge to successful staged wound closure without flap reconstruction.
- A tailored three-layer interface may enhance local infection control while preserving the vascular graft.

Over the last decade, negative pressure wound therapy (NPWT) has been increasingly adopted in selected cases of vascular graft infection or exposure^[^3^]^, although its use over exposed vascular structures remains controversial because of the perceived risk of catastrophic hemorrhage^[^4^]^ and evidence regarding its use in cases with exposed anastomotic sites and a complete absence of reconstructive options remains extremely limited.

This report describes the successful salvage of an exposed and infected limb of an aorto-bifemoral graft using low-pressure NPWT with a multilayer protective interface, employed as a bridging therapy within a staged wound closure strategy in a patient with no viable reconstructive options. This case report has been prepared in line with the SCARE checklist^[^5^]^.

## Case presentation

A 71-year-old man with a previous prosthetic aorto-bifemoral bypass graft and a left transtibial amputation (2013) was admitted with a rupture of a left femoral anastomotic pseudoaneurysm causing acute ischemia of the ipsilateral stump. Preoperative CT angiography demonstrated a large pseudoaneurysm resulting from detachment of the left femoral limb of the prosthetic bypass (Fig. 1).

**Figure 1.**
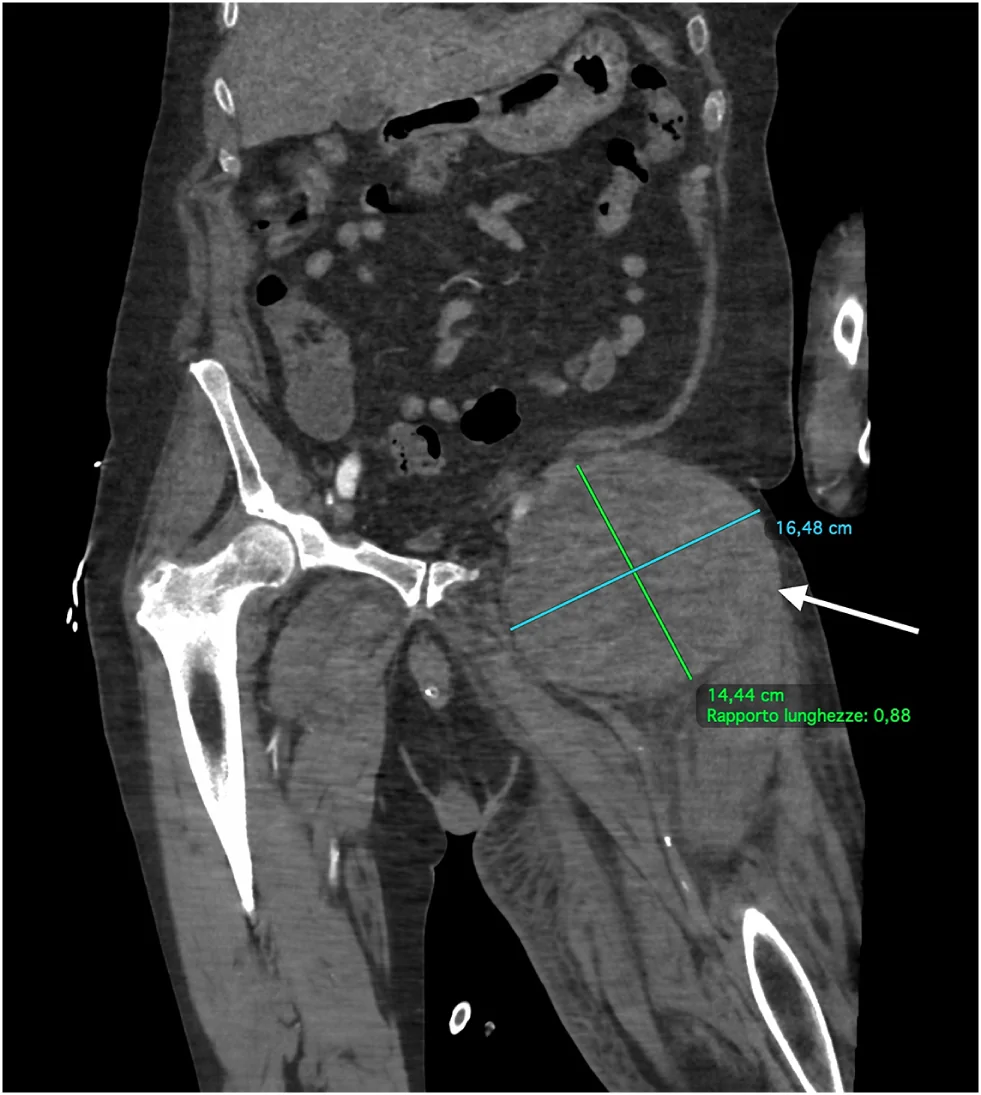
Preoperative CT angiography demonstrates a large pseudoaneurysm (arrow) caused by disruption of the distal anastomosis of the left limb of the aorto-bifemoral bypass.

The patient underwent emergency open surgical revision with evacuation of a large hematoma and reconstruction of the arterial axis. Given the chronic occlusion of the superficial femoral artery, distal revascularization was achieved through the profunda femoris artery using a Dacron interposition graft.

Following vascular reconstruction, an extensive hematoma and soft-tissue necrosis required aggressive debridement. This resulted in severe damage to the regional musculature and a large inguino-femoral soft-tissue defect (approximately 15 × 30 cm), with exposure of the vascular graft and distal anastomotic sites (Fig. 2).

**Figure 2.**
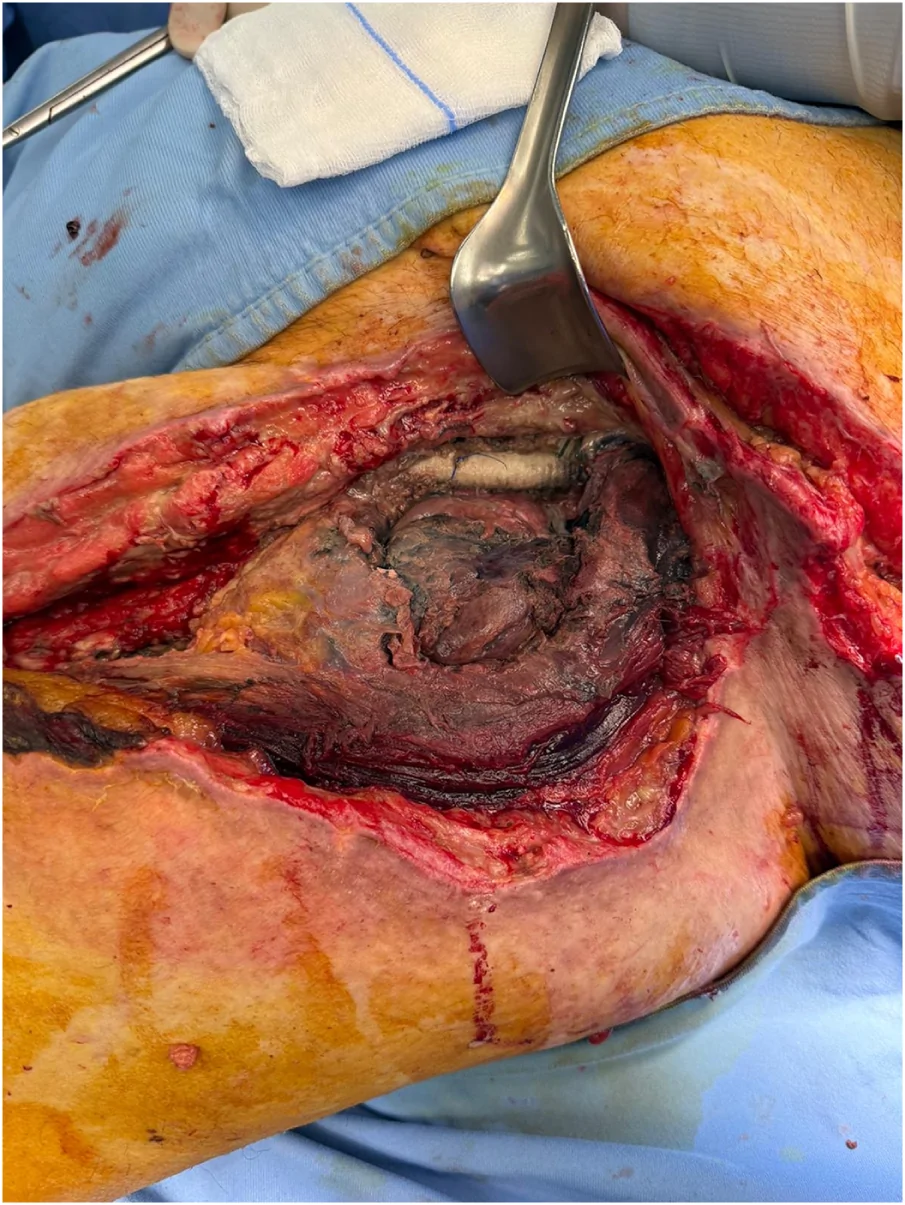
Extensive inguino-femoral wound dehiscence with exposure of the vascular graft and anastomotic sites following aggressive surgical debridement. A surgical retractor was used to improve visualization of the exposed graft for documentation purposes; despite the apparent soft-tissue redundancy under traction, tension-free primary closure was not feasible because of the extensive soft-tissue defect.

The postoperative course was complicated by multi-organ failure (MOF) requiring temporary renal replacement therapy. Microbiological cultures also revealed Enterobacter cloacae, followed by a bloodstream infection due to extended-spectrum beta-lactamases-producing strains, requiring targeted antibiotic therapy with meropenem. Due to persistent graft exposure and the complexity of the defect, a multidisciplinary approach involving vascular surgery, plastic surgery, and infectious disease specialists was established.

A temporary NPWT strategy was initiated to isolate the graft from external contamination while awaiting clinical stabilization and definitive reconstructive planning. NPWT was applied using a protective multilayer interface composed of an antimicrobial non-adherent dressing (Acticoat Flex 3^®^) placed directly over the graft to provide local antimicrobial activity (Fig. 3), followed by a layer of white polyvinyl alcohol (PVA) foam to minimize mechanical stress on the underlying structures, and an outer layer of black polyurethane foam to complete the negative pressure system. Negative pressure was set at −75 mmHg.

**Figure 3.**
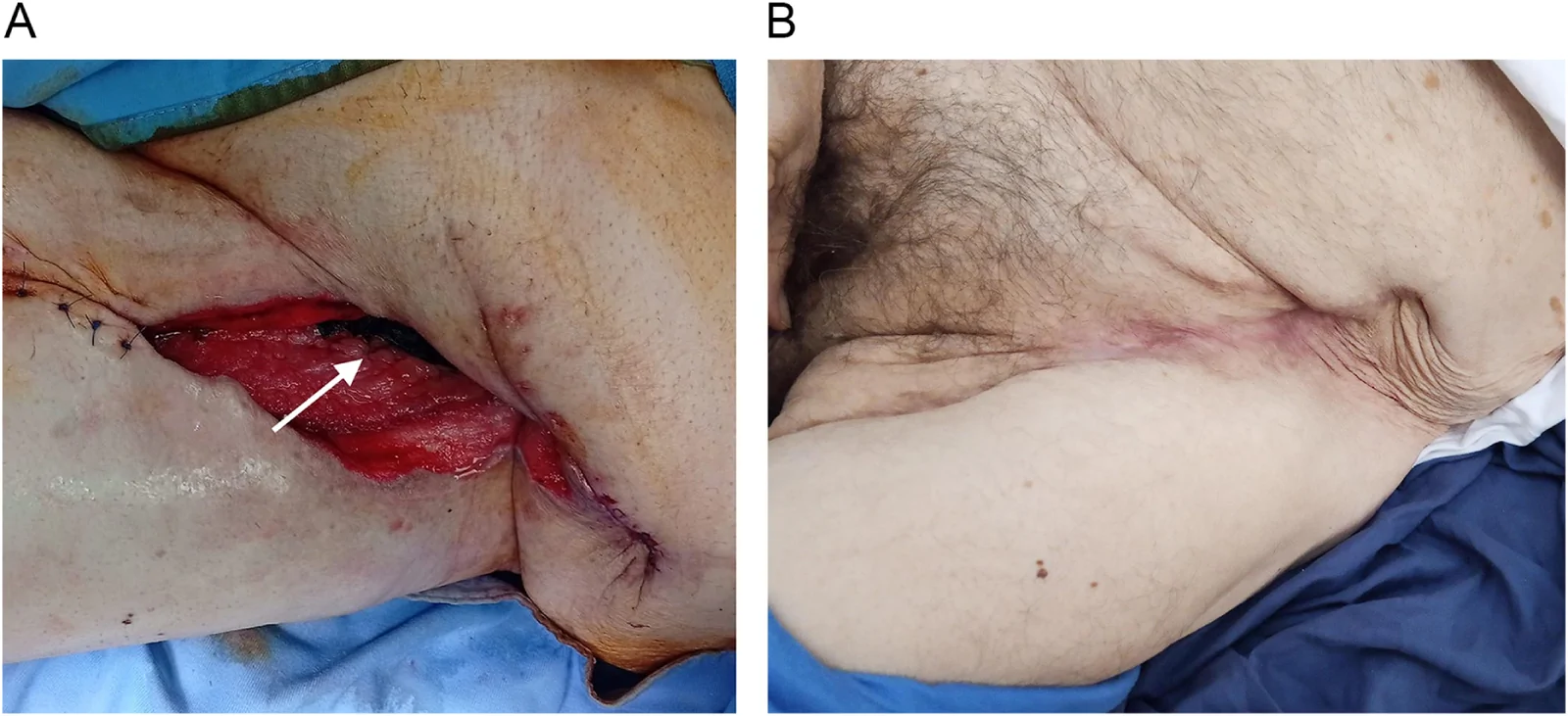
(A) Intraoperative photograph showing the antimicrobial non-adherent dressing (Acticoat Flex 3^®^; white arrow) placed directly over the exposed vascular graft as part of the customized three-layer NPWT interface. (B) Clinical appearance at the 5-month follow-up, showing complete wound healing with no evidence of recurrent infection.

Because of the risk of catastrophic hemorrhage, treatment was performed under close inpatient surveillance in the vascular surgery unit.

After recovery from MOF, the patient was reassessed by the multidisciplinary team. Graft explantation with autologous venous reconstruction was considered but rejected because of the absence of a suitable venous conduit and the patient’s physiological frailty. Contrast-enhanced CT angiography demonstrated occlusion of the contralateral deep inferior epigastric artery, excluding a vertical rectus abdominis myocutaneous flap or a vertical pedicled deep inferior epigastric perforator flap reconstruction. In addition, the main vascular pedicles of the gracilis and tensor fasciae latae muscles were unavailable, while advanced peripheral arterial disease and profunda-dependent limb perfusion precluded free tissue transfer. Combined with the extensive post-debridement soft-tissue loss, these findings established a true “no-option” reconstructive scenario.

A staged wound closure strategy with progressive approximation of the wound edges was therefore performed during weekly NPWT dressing changes.

Complete wound closure was achieved 69 days after the initiation of NPWT. The patient remained hospitalized for an additional month before discharge. At the 5-month follow-up, no clinical signs of recurrent infection were observed (Fig. 3). A timeline of the patient’s clinical course is provided in Table 1.

**Table 1:** Timeline of the patient’s clinical course.

Timeline	
time point	Clinical event
Before presentation	Aorto-bifemoral bypass for advanced peripheral arterial disease
Day 0	Rupture of the distal anastomosis of the left bypass limb with femoral pseudoaneurysm and acute stump ischemia
Day 0	Emergency vascular reconstruction with Dacron interposition graft to the profunda femoris artery
Day 1	MOF requiring continuous renal replacement therapy
Day 14	Transition from continuous to intermittent dialysis
Day 27	Extensive debridement and initiation of low-pressure NPWT with three-layer protective interface
Days 33–52	Serial debridement and weekly NPWT dressing changes
Day 59	Initiation of staged wound closure during NPWT changes
Day 68	CT angiography confirmed a true “no-option” reconstructive scenario
Day 69–90	Progressive staged wound closure during weekly NPWT changes
Day 96	Complete wound closure achieved
Day 126	Hospital discharge

## Discussion

Management of exposed and infected vascular grafts remains challenging because the use of NPWT over exposed grafts and anastomotic sites is still limited by concerns about the risk of catastrophic hemorrhage. Nevertheless, Monsen *et al* demonstrated significantly faster wound healing with NPWT than with alginate dressings in patients with deep perivascular groin infections (57 vs. 104 days; *P* = 0.026), without increasing major complications^[^6^]^. Similarly, several retrospective case series have reported graft preservation in the majority of patients with acceptable complication rates when NPWT was combined with protective interfaces^[^3,7–12^]^. In the largest published series, Andersson *et al* reported major bleeding in approximately 7% of patients, although these events were considered more closely related to the underlying vascular pathology than to NPWT itself^[^10^]^.

More recently, customized low-vacuum protocols (<80 mmHg) have been proposed specifically for high-risk patients with complex wounds over exposed vessels, reporting encouraging preliminary outcomes while emphasizing the need for further validation^[^13^]^.

Against this background, our case extends the current evidence in two clinically relevant respects.

First, unlike previous reports using either a non-adherent interface or PVA foam alone, we combined three complementary components with distinct functions. A silver-impregnated non-adherent dressing (Acticoat Flex 3^®^) was placed directly over the exposed graft and anastomotic sites to provide local antimicrobial activity while protecting the graft from mechanical trauma. This was covered by a PVA foam layer to minimize adherence and shear stress, followed by an outer polyurethane foam layer to complete the NPWT system. To our knowledge, the deliberate addition of an antimicrobial interface directly over an exposed vascular graft has not been previously described. This approach is supported by Monsen *et al*, who found that NPWT alone did not improve bacterial clearance^[^6^]^.

Second, the originality of this case lies in the reconstructive setting rather than in the use of NPWT itself. Unlike in previous reports, flap reconstruction was not feasible, making staged direct wound closure the only reconstructive option. The absence of other reconstructive options made NPWT, followed by staged wound closure, the only feasible limb- and graft-preserving strategy. This “no-option” scenario is rarely documented in the literature and has direct implications for reconstructive decision-making.

The presence of exposed anastomotic sites further increased the theoretical risk of catastrophic bleeding. Therefore, continuous low-pressure NPWT (−75 mmHg) was selected, consistent with low-vacuum protocols^[^13^]^, to minimize mechanical stress on the vascular structures while preserving the biological effects of NPWT. Close monitoring during dressing changes and throughout hospitalization was essential to ensure patient safety.

Failure to preserve the graft would likely have resulted in hip disarticulation.

Although limited by its single-case design and short follow-up, this report describes a true “no-option” reconstructive scenario and a customized three-layer NPWT interface that may help guide management in similar patients.

## Conclusion

Although our findings cannot be generalized, a tailored three-layer protective interface incorporating a silver-impregnated dressing may enhance local infection control in exposed vascular grafts. In carefully selected patients, low-pressure NPWT followed by staged wound closure may represent a valuable limb- and graft-preserving strategy. Further studies are required to define the reproducibility of this approach.

## Data Availability

Not applicable.

## References

[1] Wilson WR Bower TC Creager MA. Vascular graft infections, mycotic aneurysms, and endovascular infections: a scientific statement from the American heart association. Circulation 2016;134:e412–e460. 10.1161/CIR.0000000000000457 27737955

[2] Colwell AS Donaldson MC Belkin M. Management of early groin vascular bypass graft infections with sartorius and rectus femoris flaps. Ann Plast Surg 2004;52:49–53. 10.1097/01.sap.0000099960.68038.53 14676699

[3] Pinocy J Albes JM Wicke C. Treatment of periprosthetic soft tissue infection of the groin following vascular surgical procedures by means of a polyvinyl alcohol-vacuum sponge system. Wound Repair Regen 2003;11:104–09. 10.1046/j.1524-475x.2003.11205.x 12631297

[4] Mendez-eastman S. Guidelines for using negative pressure wound therapy. Adv Skin Wound Care 2001;14:314–22. 10.1097/00129334-200111000-00015 11794443

[5] Kerwan A Al-jabir A Mathew G. Revised Surgical CAse REport (SCARE) guideline: an update for the age of Artificial Intelligence. Prem J Sci 2025;10:100079.

[6] Monsen C Wann-hansson C Wictorsson C. Vacuum-assisted wound closure versus alginate for the treatment of deep perivascular wound infections in the groin after vascular surgery. J Vasc Surg 2014;59:145–51. 10.1016/j.jvs.2013.06.073 24055512

[7] Svensson S Monsen C Kölbel T. Predictors for outcome after vacuum-assisted closure therapy of perivascular surgical site infections in the groin. Eur J Vasc Endovasc Surg 2008;36:84–89. 10.1016/j.ejvs.2007.12.020 18294875

[8] Mayer D Hasse B Koelliker J. Long-term results of vascular graft and artery preserving treatment with negative pressure wound therapy in Szilagyi grade III infections justify a paradigm shift. Ann Surg 2011;254:754–60. 10.1097/SLA.0b013e3182365864 21997817

[9] Acosta S Monsen C. Outcome after VAC therapy for infected bypass grafts in the lower limb. Eur J Vasc Endovasc Surg 2012;44:294–99. 10.1016/j.ejvs.2012.06.005 22818802

[10] Andersson S Monsen C Acosta S. Outcome and complications using negative pressure wound therapy in the groin for perivascular surgical site infections after vascular surgery. Ann Vasc Surg 2018;48:104–10. 10.1016/j.avsg.2017.10.018 29217444

[11] Cheng HT Hsu YC Wu CI. Efficacy and safety of negative pressure wound therapy for Szilagyi grade III peripheral vascular graft infection. Interact Cardiovasc Thorac Surg 2014;19:1048–52. 10.1093/icvts/ivu289 25185571

[12] Berger P de Bie D Moll FL. Negative pressure wound therapy on exposed prosthetic vascular grafts in the groin. J Vasc Surg 2012;56:714–20. 10.1016/j.jvs.2012.02.007 22554424

[13] Paolini G Sorotos M Firmani G. Low-vacuum negative pressure wound therapy protocol for complex wounds with exposed vessels. J Wound Care 2022;31:78–85. 10.12968/jowc.2022.31.1.78 35077217

